# Practices and Policies Around Wellness: Insights From the Internet Crimes Against Children Task Force Network

**DOI:** 10.3389/fpsyt.2022.931268

**Published:** 2022-06-20

**Authors:** Kimberly J. Mitchell, Ateret Gewirtz-Meydan, Jennifer O'Brien, David Finkelhor

**Affiliations:** ^1^Crimes Against Children Research Center, University of New Hampshire, Durham, NH, United States; ^2^Faculty of Social Welfare and Health Sciences, School of Social Work, University of Haifa, Haifa, Israel; ^3^Department of Social Work, University of New Hampshire, Durham, NH, United States

**Keywords:** wellness, internet crimes against children, law enforcement, child sexual abuse material, vicarious trauma

## Abstract

This article aims to understand what practices and training Internet Crimes Against Children (ICAC) Task Forces and affiliated agencies are currently using to help mitigate distress and promote wellbeing among investigators of child sexual abuse material (CSAM). Data were collected via telephone interviews with Commanders of 54 ICAC Task Forces as well as an online survey of 155 investigators at ICAC-affiliated agencies. Sixty-two percent of respondents said their agency had an Officer Wellness Program. Findings highlighted considerable gaps in protective practices with 46.1% of respondents mentioning the need for more wellness resources in their agency for personnel who have viewed CSAM as a high priority. Stigma created by help-seeking was the most widely acknowledged barrier discussed in relation to police wellness. A large and salient problem was the persistent lack of wellness practices in the affiliated agencies in comparison to the Task Forces themselves. Exposure to CSAM can be a source of stress for personnel and the results indicate concern about the problem and a diffusion of proactive initiatives, but barriers and inconsistent adoption remain.

## Introduction

Since the expansion of the Internet in the mid-1990s, law enforcement is encountering a growing number of cases that involve the possession, distribution, and production of child pornography (1); more commonly known as child sexual abuse material (CSAM), including both images and videos. Since these cases involve technology, their handling requires the involvement of forensic examiners and investigators with specialized technical expertise and equipment. Consequently, many CSAM investigations are handled by specialized law enforcement units, such as the Internet Crimes Against Children (ICAC) Task Forces [https://www.icactaskforce.org/]. The ICAC Task Force Program is a national network of 61 coordinated Task Forces, representing ~5,000 affiliated federal, state, and local law enforcement agencies dedicated to investigating, prosecuting and developing effective responses to internet crimes against children. ICAC Task Force agencies all receive some level of federal funding whereas the affiliated agencies do not receive any direct federal funding. As such, affiliated agencies are reliant upon receiving funding, training, and resources from one of the 61 Task Force agencies or obtaining their own funding for ICAC Program work.

In the course of handling cases of CSAM, ICAC and affiliated personnel may be exposed to considerable quantities of material that graphically portray rape and child sexual abuse (2). These images can be extremely disturbing because they violate strongly held standards of ethical behavior and depict egregious, offensive, and disturbing acts involving child victims. Research from the CyberTipline, operated by the National Center for Missing & Exploited Children (3), estimates that nearly one million CSAM images and videos were being flagged on the Internet each month by law enforcement in 2017, and the rate has been growing at 51% per year (1). There is extensive concern among law enforcement that viewing such material may have corrosive effects on investigators' mental health, particularly resulting in symptoms of traumatic stress (4, 5).

### Child Sexual Exploitation Investigations and Secondary Trauma

Researchers have determined that traumatic stress rates among law enforcement are already high compared to other citizens (6, 7). However, most of this research has been conducted with line officers who investigate street crimes with primarily physical risks. Less research has been conducted on the impact of traumatic stress among officers on cybercrime and digital forensic units. Given their exposure to CSAM and child sexual exploitation cases, there is a significant risk of secondary trauma for these investigators (8). Indeed, research has shown that crimes involving child victims are particularly distressing because investigators may have difficulty creating emotional distance between themselves and victims, identify with victims, think of their own children as victims, or feel they or society have failed to protect victims (6, 9–12).

Traumatic stress symptoms include intrusive thoughts, avoidance behaviors, changes in cognition and mood, and emotional arousal, as well as depression, substance abuse and somatic complaints (13). Investigators of technology-facilitated child exploitation have reported experiencing these post-traumatic stress symptoms, as well as discomfort interacting with children, reduced emotional and physical intimacy with partners, and a heightened awareness of the potential presence of child sex abusers in their social networks (2, 14–16). Law enforcement investigators who also conducted digital CSAM forensic exams scored significantly higher on secondary traumatic stress, higher on feelings of worthlessness, and lower on concentration compared to digital forensic examiners who were not investigators in one study (5). Somatic complaints are also noted and include headaches, intestinal upsets, severe tiredness, sleep deprivation, depressed immune response, nausea, elevated heart rate and general ill health (14, 16, 17). Social problems include reluctance to interact with children, withdrawal from social activities with family, friends or partners, negative opinions of other people, isolation from other law enforcement personnel and decreased desire for physical/emotional intimacy within marital relationships (14–16, 18, 19). Investigators also describe how they “switch off” at the end of the day, distancing themselves not only from their partner but also from the routine physical interaction with their own children (16).

Even with preliminary research connecting CSAM investigations with elevated stress, there are other factors that may influence or even offset traumatic stress (2). A number of studies have found that organizational factors were more influential in explaining police stress than traumatic job-related exposures (20–22). Agency rules and regulations, departmental culture and how supervisors interact with personnel may be more important than job duties or critical incidents. Moreover, personnel required to work with CSAM may have particular organizational frustrations. For example, they may lack up-to-date computer equipment and ready technical support (23). The culture of an agency may also be a factor. Stress could be exacerbated for personnel who are subjected to inappropriate, minimizing, or otherwise invalidating comments from colleagues about CSAM and the psychological impact it may cause, for example.

Some departmental policies, investigative practices and even some personal characteristics of investigators could also foster resilience to such stress reactions (24). Indeed, in one study, when the organizational climate was perceived to be positive, investigators were less adversely affected by investigating internet child abuse material (2). A positive organizational climate included features like job satisfaction, minimal role overload, higher levels of work engagement, pride in the unit, and respect from other units. Other measures such as allowing special leaves, creating investigator support groups, and teaching stress reduction techniques may also be helpful. Generic police programs have been developed to teach resilience skills (25); the identification of such resiliency factors in regard to CSAM exposure would aid in the development of effective policy, prevention and intervention programs for agencies.

### Officer Wellness Programs and Training

One approach to enhance the wellbeing among law enforcement personnel is the utilization of Officer Wellness Programs. These programs vary across agencies, but typically include comprehensive strategies to promote safety and wellness by addressing topics such as physical fitness, mental health, emotional wellness, stress management, financial wellness, peer support, and family support, as well as tactical and operational safety considerations. Yet, in a study conducted over a decade ago among 40 ICAC Task Forces and 471 affiliate agencies, only 13% of ICAC Task Forces and 5% of affiliates had mandatory mental health requirements (19). Interestingly, while only 4% of surveyed agencies reported no mental health resources, 39% of participants reported their agencies needed more such resources for personnel exposed to CSAM.

Currently, the vast majority of organizations, including the International Association of Chiefs of Police (26), recognize the need for more mental health services and the importance of creating a healthy work environment for investigators of child exploitation cases, to limit traumatic stress. The Innocent Justice Foundation, an Office of Juvenile Justice and Delinquency Prevention ICAC National Training Program funded project, developed the Supporting Heroes in Mental Health Foundational Training (SHIFT) wellness program in 2008 to train mental health professionals and provide resources to assist law enforcement officers exposed to CSAM (see http://www.shiftwellness.org). The SHIFT wellness program provides recommendations for supervisors to create a healthy and supportive work environment, and to avoid burnout, compassion fatigue, and traumatic stress among personnel. These work environment recommendations include work flexibility and proper preparation of new employees. Although finding qualified mental health providers is strongly encouraged, there is no mention of whether psychological evaluation should be required, since resources may be available but underutilized by law enforcement due to stigma.

### Current Study

Since awareness has been growing and new programs are becoming available, it is important to track progress in providing help to this segment of the law enforcement community. The current study sought to understand what practices and training ICAC Task Forces and affiliated agencies are currently using to help mitigate distress and promote wellbeing among CSAM investigators and to identify characteristics of agencies which are implementing more proactive wellness efforts. Given the more specific role ICAC Task Forces play in the investigation of CSAM compared with their affiliated agencies, which are varied in size and structure, the research questions listed below will compare these two separate but related types of agencies. Specifically, we address the following research questions: (1) What department personnel are exposed to CSAM and how are they chosen for the position? (2) What percentage of ICAC Task Forces and affiliate agencies have an Officer Wellness Program? What other strategies do agencies use to promote well-being among personnel? (3) What agency characteristics are related to engagement in more proactive efforts around wellness?

## Materials and Methods

### Participants

Data were collected as part of a larger study on police wellness among investigators of child sexual abuse material. Participants for the current paper consisted of (1) Commanders of the 61 Internet ICAC Task Forces and (2) a purposive sample of investigators at ICAC-affiliated agencies. Of the 61 Commanders, 54 (89%) participated in telephone interviews about agency-level policies and practices around police wellness. ICAC affiliated agencies were recommended for participation by ICAC commanders. Of the 351 investigators from affiliated agencies invited to participate, 17 could not be contacted and 15 were duplicate email addresses; of the 339 eligible email addresses, 155 (46%) completed a parallel online survey representing 151 unique affiliated agencies. Nineteen percent of all respondents were female. Most participants held titles such as investigator, lieutenant, officer, detective, or sergeant and, as such, we use the term “investigator” to describe them in the current manuscript. No additional demographic characteristics were gathered.

### Procedures

Trained research assistants conducted telephone interviews with ICAC commanders between September and December 2020. The interviewers attended a training session that provided extensive details about the background, purpose and instrumentation of the study, and they participated in practice interviews before beginning data collection. At the end of the interview Commanders were asked to nominate investigators at some of their affiliated agencies to complete the parallel online survey; email addresses were provided by Commanders.

The email invitation to affiliated agency investigators explained the purpose of the survey, informed potential participants that their responses were confidential, and that participation was voluntary. The online surveys were conducted using Qualtrics, an online survey service that enables the creation of survey instruments, distribution of surveys, data collection, and storage. Two reminder emails were sent at ~2-week intervals for participants who had not completed the online survey. The data collection period for the online affiliated agency surveys was December 2020 to February 2021. At the beginning of the survey, affiliated agency participants were told that they could pause the survey and start again later when it was a better time for them and given instructions on how to do this. ICAC Commanders were similarly told they could stop the interview at any time and skip any questions they did not want to answer. All data were collected under the oversight of the University of New Hampshire Institutional Review Board.

### Measures

The survey instrument was developed specifically for this study. Questions were designed through interviews and consultations with criminal justice personnel and mental health providers. All questions were the same for both ICAC Commander interviews and surveys with investigators in affiliated agencies. These questions covered several different aspects of employee wellness including who in the department has exposure to CSAM, the preparation personnel receive prior to first exposure, and qualifications/requirements for the position. Items also queried the existence of an Officer Wellness Program, discussion of signs of stress during staff meetings or training sessions, and other options for employees that could help promote wellness. Respondents were also asked if there was a police union in their agency that supported wellness. This was coded into two variables for analyses: union supportive of wellness (vs. all other) and no union (vs. all other). Open-ended questions asked about different needs and challenges around wellness in their agency. Specifically, participants were asked: (1) What other criteria are being used to pick personnel to work CSAM cases? (*n* = 113 responses); (2) What is working best about your Officer Wellness Program? (*n* = 91 responses); (3) Do you have any concerns about having an Officer Wellness Program? (*n* = 11 responses); (4) Have you encountered any barriers when implementing your wellness program? (*n* = 27 responses); (5) How do you think your [wellness] strategies could be improved? (*n* = 124 responses); and (6) Overall, what would you say are the three most successful things your agency does to promote wellness? (*n* = 132 responses).

Finally, we identified four variables we felt were suggestive of more proactive efforts to promote wellness within the agency: (1) the existence of an Officer Wellness Program (yes/no), (2) providing preparation to personnel before exposure to CSAM (yes/no), (3) screening personnel for child abuse and neglect experience before assignment to CSAM investigations (yes/no), and (4) having at least one agency-level support policy around CSAM (yes/no). This final support policy variable was coded as yes if the agency had at least one of the following in place: staff meetings where reactions to CSAM are discussed; group or individual sessions led by a mental health professional where reactions to CSAM are discussed; individual case reviews where reactions are discussed; rotations or time limits on positions that require viewing CSAM; part-time assignments or the ability to pursue other aspects of law enforcement; exit tickets which allow sworn personnel to transfer with no questions asked or penalties; exit interviews or debriefings for personnel who leave positions requiring viewing CSAM; and follow-up contact to check on personnel who have left positions requiring viewing of CSAM.

### Data Analysis

First, we reported on the types of department personnel who work on CSAM investigations and the types of preparation personnel are given before they are first exposed. Differences between ICAC Task Force agencies and affiliated agencies were explored using chi-square statistics. Then, we compared reports of having an Officer Wellness Program and other strategies for promoting well-being across Task Forces and affiliated agencies using chi-square statistics. Differences between ICAC Task Forces and affiliated agencies were examined for needs and challenges around employee wellness. Throughout, qualitative variables were included to provide context and nuance to the quantitative responses. For these, qualitative coding of the open-ended responses was conducted using an inductive process of content analysis with codes developed by the second and third authors following guidelines for qualitative coding (27, 28). All responses were coded by the second and third author; overall, inter-rated reliability was strong. Coding discrepancies were discussed between raters and agreed upon prior to the final analysis. Finally, four logistic regression analyses were conducted representing each of the different proactive efforts around wellness described earlier as outcomes and both agency- and participant-level characteristics serving as independent variables. For each outcome, unadjusted odds ratios for each agency and participant characteristic were provided and then, a final parsimonious logistic regression was conducted inclusive of those characteristics significant at *p* < 0.05 or better in the unadjusted analyses.

## Results

### Department Personnel Exposed to CSAM

Across all respondents, 91.9% said that police officers/investigators in their agencies were exposed to CSAM during investigations with no differences between ICAC and affiliated agencies (Table 1). Forensic examiners who were sworn staff (65.1%) and forensic examiners who were civilian (23.4%) were also exposed to CSAM material with higher percentages of each noted by ICAC Task Forces compared to affiliated agencies. Some respondents also said that administrative staff were exposed to CSAM during investigations (15.8% overall) with more ICAC Task Forces indicating this (53.7%) compared to affiliated agencies (2.6%) (*p* < 0.001). Over half (57.4%) of participants said personnel are given some preparation before first exposure to CSAM with this being more common among ICAC Task Forces (85.2%) than affiliated agencies (47.7%). Examples of such preparation included discussion or education on the impact of work-related CSAM exposure (89.2%), mention of support availability (88.3%), and exposure to examples of CSAM (45.8%). Seven in ten (70.2%) respondents said that personnel chosen to conduct CSAM investigations were given the choice to opt out once they have seen some of the material; this was more common among ICAC Task Forces vs. affiliated agencies (*p* = 0.002). Over half (53.4%) said that potential CSAM personnel were screened for whether they had prior experience working child abuse and neglect cases while 10.1% said it was a requirement to have this background. Nine percent of respondents said personnel were given mental health testing or screening before they began this work.

**Table 1:** Department personnel who work CSAM investigations.

	**Combined (*n =* 209) % (*n*)**	**ICAC affiliate agencies** **(*n =* 155) % (*n*)**	**ICAC Task Forces** **(*n =* 54) % (*n*)**	***P*** **-value**
**Who in agency has exposure to CSAM during investigations**				
Police officers/Investigators	91.9 (192)	92.9 (144)	88.9 (48)	0.35
Forensic examiners who are sworn staff	65.1 (136)	56.1 (87)	90.7 (49)	<0.001
Forensic examiners who are civilian	23.4 (49)	14.2 (22)	50.0 (27)	<0.001
Administrative staff	15.8 (33)	2.6 (4)	53.7 (29)	<0.001
Other staff	12.0 (25)	6.5 (10)	27.9 (15)	<0.001
**Personnel given preparation before they are first exposed to CSAM (any)**	57.4 (120)	47.7 (74)	85.2 (46)	<0.001
Exposure to examples of CSAM	45.8 (55)	37.8 (28)	58.7 (27)	0.03
Discussion or education on the impact of work-related CSAM exposure	89.2 (107)	85.1 (63)	95.7 (44)	0.07
Mention of support availability	88.3 (106)	82.4 (61)	97.8 (45)	0.01
**Other CSAM-specific activities**	(*n =* 209)	(*n =* 155)	(*n =* 54)	
Given option to self-select out once they have seen some of the material	70.2 (146)	64.3 (99)	87.0 (47)	0.002
Screened for or asked whether they have histories of working child abuse and neglect cases	53.4 (111)	45.5 (70)	75.9 (41)	<0.001
Requirement to have worked CAN cases before beginning to work CSAM cases	10.1 (21)	13.0 (20)	1.9 (1)	0.02
New personnel asked what they do to relieve stress	48.3 (101)	40 (62)	72.2 (39)	<0.001

*CSAM, child sexual abuse material; CAN, child abuse and neglect*.

Open-ended responses regarding other criteria agencies were using to select personnel to work CSAM cases fell into five broad categories: (1) Experience; (2) Willingness; (3) Mental Health and Resilience; (4) Personality Traits and Characteristics; and (5) Departmental Culture. Specifically, 80 respondents (70.8% of those who provided a qualitative response) reported that “Experience” was one of the top criteria used to select personnel. Experience was defined as any prior working experience relevant to CSAM investigations. In the words of one respondent, “[We look for] forensic training, education, experience in the field of forensics, experience with cell phones, investigative experience. You are usually hired as a detective before you come to our unit.” Other types of experience mentioned included technology generally, social media, and crimes against children.

Thirty-four respondents (30% of respondents to this question) reported that “Willingness” to work the job, despite the emotional weight of the issue and other departmental tasks, was an important criterion for selection to work CSAM cases. This included a “willingness to work these types of cases” as well as “a desire to learn.” The ’Mental Health and Resilience' of potential hires was mentioned by 9 respondents (8% of respondents to this question). Notably, these respondents reported asking about lives outside of work, support structures, and coping skills. In the words of one participant, “The commander asks how they handle stress and other questions to make sure they are appropriate for this type of work”.

Beyond the specifics of mental health and resilience, 21 respondents (18.6% of respondents to this question) reported more general ’Personality Traits and Characteristics' that they deemed important for this work. In many cases these characteristics were somewhat vague. In a statement echoed by many, one respondent wrote “I actively recruit investigators that I believe would do a great job and can handle the work.” Other respondents had more specific characteristics: “I tend to select investigators who don't have daughters. They seem to last longer in the field.” Finally, 14 respondents (12.4% of respondents to this question) mentioned the importance of new personnel fitting into the existing ’Departmental Culture.' In the words of one respondent: “They need to fit in with the current staff”.

### Officer Wellness Programs

Sixty-two percent of respondents said their agency had an Officer Wellness Program with no differences noted between ICAC Task Forces and affiliated agencies (Table 2). Many of these were agency-funded (64.3%) while some were ICAC grant-funded (24.8%), and state-funded (10.1%). More ICAC Task Forces indicated ICAC grant funding while more affiliated agencies indicated agency funding. One in four (25.6%) said that participation in the program was mandatory. Among respondents for ICAC Task Forces, 41.7% said their Officer Wellness Program was made available to affiliated agencies (22.2% were not sure).

**Table 2:** Officer Wellness Programs and other strategies for well-being.

	**Combined (*n =* 209) % (*n*)**	**ICAC Affiliate Agencies** **(*n =* 155) % (*n*)**	**ICAC Task Forces** **(*n =* 54) % (*n*)**	***P*** **-value**
**Officer Wellness Programs**				
Currently have an Officer Wellness Program	62.0 (129)	60.4 (93)	66.7 (36)	0.41
ICAC grant funded	24.8 (32)	18.3 (17)	41.7 (15)	0.006
Agency funded	64.3 (83)	71.0 (66)	47.2 (17)	0.01
State funded	10.1 (13)	8.6 (8)	13.9 (5)	0.37
Participation is mandatory	25.6 (33)	22.6 (21)	33.3 (12)	0.21
Make available to affiliate agencies			41.7 (15)	
Not sure	—	—	22.2 (8)	—
**Staff meetings or training session discussion topics**				
Signs of stress at work	34.5 (72)	20.7 (32)	74.1 (40)	<0.001
Signs of stress at home	32.1 (67)	19.3 (30)	68.5 (37)	<0.001
Relationship problems	27.3 (57)	14.2 (22)	64.8 (35)	<0.001
Sexual problems	16.7 (35)	9.0 (14)	38.9 (21)	<0.001
**Policies or resources available in agency**				
Staff meetings where reactions to CSAM are discussed	33.5 (70)	21.3 (33)	68.5 (37)	<0.001
Group or individual session led by a mental health professional where reactions to CSAM are discussed	28.7 (60)	19.3 (30)	55.6 (30)	<0.001
Individual case reviews where reactions are discussed	32.1 (67)	25.2 (39)	51.9 (28)	<0.001
Rotations or time limits on positions that require viewing CSAM	12.4 (26)	11.0 (17)	16.7 (9)	0.27
Part-time assignments or the ability to pursue other aspects of law enforcement	29.7 (62)	16.8 (26)	66.7 (36)	<0.001
Exit tickets which allow sworn personnel to transfer with no questions asked or penalties	31.1 (65)	18.1 (28)	68.5 (37)	<0.001
Exit interviews or debriefings for personnel who leave positions requiring viewing CSAM	23.4 (49)	11.6 (18)	57.4 (31)	<0.001
Follow-up contact to check on personnel who have left positions requiring viewing of CSAM	14.3 (30)	7.7 (12)	33.3 (18)	<0.001
**Agency opportunities**				
Offers ample vacation/personal time off	83.3 (174)	78.7 (122)	96.3 (52)	0.003
Provides daily opportunities for CSAM investigators to debrief with other CSAM investigators	48.3 (101)	31.6 (49)	96.3 (52)	<0.001
Provides regular administrative updates about positive outcomes from CSAM investigations	41.6 (87)	25.2 (39)	88.9 (48)	<0.001
**Other options to help promote wellness**				
Cafeteria with healthy meal options	9.6 (20)	7.7 (12)	14.8 (8)	0.13
Onsite gym	63.6 (133)	65.8 (102)	57.4 (31)	0.27
Outdoor grounds for walking, running or sitting to eat	53.6 (112)	41.9 (65)	87.0 (47)	<0.001
Indoor workspaces that are sunny and comfortable	32.5 (68)	19.3 (30)	70.4 (38)	<0.001
Options to bring your own dog to work	8.6 (18)	3.2 (5)	24.1 (13)	<0.001
Police dog on premises	28.7 (60)	26.5 (41)	35.2 (19)	0.22
Employee Assistance Program	76.6 (1,600)	71.6 (111)	90.7 (49)	0.004
Peer counselors	46.9 (98)	41.3 (64)	63.0 (34)	0.006
Chaplains, including volunteer chaplains	64.1 (134)	59.3 (92)	77.8 (42)	0.01
Other	10.5 (22)	5.2 (8)	25.9 (14)	<0.001

*CSAM, child sexual abuse material*.

Only nine respondents (4%) with an Officer Wellness Program told us about strategies and services their program did not offer but were frequently requested. These strategies included massage therapy, mandatory mental health check ins, family supports (e.g., mental health resources for spouses and family members), and more community referral sources. A few (*n* = 4) respondents noted that their staff has asked for an occasional team building day. In the words of one respondent, “They have requested…a day a month to “bond” outside of the office setting in a ’fun activity' to clear the mind”.

A minority (5.3%, *n* = 11) of respondents offered concerns about having such a wellness program. Specifically, individuals noted concerns about “Funding” (e.g., “The value of the program isn't always seen by the administrators since it's funded by ICAC and not out of the general fund”). Respondents described having limited amounts of funding, and noted that funding was not always consistent or available: “Initially the barrier was the funding. It was always the last thing to be funded. We would have loved to implement it sooner. Once funding was available there were no longer barriers”.

One of the main barriers for wellness programs was related to the “Stigma on Mental Health.” As one respondent described, “I think that in policing in general we have come a long way, but we still have a ways to go. I think as a society with our views of mental health we still are struggling and we need to make this more ok and typical. Making mental health more ok is important. I try to do this in my unit.” Another respondent mentioned a desire to remove the stigma associated with the program's goal of psychological well-being (e.g., “[The commander] doesn't like the mental health label it [the wellness program] has because they tend to avoid going. [The commander] wishes it were mandatory.” Still another respondent wrote, “No one wants to be seen as someone with mental issues or be seen as crazy. Officers are often concerned about being seen as non-fit for duty.” In order to successfully implement wellness programs, respondents discussed the need to directly address the stigma and taboo around mental health: “Police by nature are very guarded and don't talk about “that stuff”. Some of the staff could be going to therapy but he [the commander] wouldn't know. If there was some way to take that taboo off the table it would be very helpful. He [the commander] isn't sure how to do that but feels communication is key”.

### Other Strategies to Promote Wellness

Respondents also told us about CSAM wellness-focused content discussed at staff meetings or training sessions in the past year. This content included: signs of stress at work (34.5%) and home (32.1%), relationship problems (27.3%), and sexual problems (16.7%). Each of these categories was more commonly endorsed by ICAC Task Forces compared to affiliated agencies (Table 2). Respondents told us about other policies or resources available in their agencies including staff meetings where reactions to CSAM were discussed (33.5%), individual case reviews where reactions were discussed (32.1%), exit tickets which allow sworn personnel to transfer with no questions asked or penalties (31.1%), part-time assignments or the ability to pursue other aspects of law enforcement (29.7%), group or individual sessions led by a mental health professional where reactions to CSAM were discussed (28.7%), and exit interviews or debriefings of personnel who leave positions requiring viewing CSAM (23.4%). Again, each of these policies and resources were more commonly endorsed by ICAC Task Forces compared to affiliated agencies. Less common policies included follow-up contact with personnel who left positions that required viewing CSAM (14.3% overall: 7.7% affiliates and 33.3% Task Forces) and rotations or time limits on positions that required viewing CSAM (12.4%).

Ninety-one respondents answered the question, “What's working best about your Officer Wellness Program?” Responses fell into four main categories including: (1) Peer Support; (2) Venting without Stigma; (3) Therapy/ Mental Health Services; and (4) Work Conditions. “Peer support” was indicated by 21 respondents (23%) whom discussed the importance of relationships among staff within the unit contributing to the wellness of each individual. This theme included employee interactions, team building, and having a group that is a “family.” Peers were described as approachable and not judgmental: “Peer support is helpful because you can just talk to them and they are easily approachable and easy to talk too.” In addition, peers were also described as people that will care for you: “The camaraderie with the staff, they are a very tight knit group. They work well together, and they can express differences openly. We have older and mature investigators; they see their job as chivalrous and we can care for each other. We can take care of each other”.

“Venting without Stigma” was discussed by 20 respondents (22%) and referred to the ability and legitimacy to talk about difficulties, personal life, and things that were deemed confidential. In the words of one respondent, “They [investigators] have created a culture within their office where it is ok to talk freely about CSAM and to talk about personal life stressors and the overwhelming workload. It is ok to talk within the agency”.

“Therapy and Mental Health Services” were also considered important and were reported by 32 respondents (35%). Offering mental health services and mandating them is in fact one of the three most successful strategies to mitigate the impact of CSAM investigations noted by respondents. The consistency of the mental health provider was also noted as important for the ability and willingness to reach out for help. As one respondent noted, “Consistency with the mental health provider [is working well for our agency]. It's been the same doctor for the last 5 years. You can develop a better relationship.” Respondents highlighted the importance of mental health services being mandatory, as some level of stigma is always associated with using mental health services. One respondent wrote, “Officers feel comfortable being able to go to someone and not having the stigma of seeking help. Especially the ones that are mandated to go, this helps with the stigma of the situation.” While respondents acknowledged the stigma and taboo associated with the use of mental health services, they also widely discussed the shift around this issue in the past years—from stigma to getting help. In the encouraging words of one respondent, “Initially law enforcement officers and support personnel were concerned about calling or asking the mental health professional for help. But now they feel comfortable to call and ask for help”.

Finally, ’Work Conditions' was the most highly reported strategy noted (*n* = 46, 50%) to increase the wellness of investigators and one of the three most effective strategies used to mitigate the impact of CSAM investigations. Work conditions included having a flexible schedule; different facilities within the agency, such as video games, a gym, cable TV access, and a large kitchen (as a place to eat and gather together); time to decompress; a varied work load; and being able to take a vacation when needed. The resources provided within the agency helped investigators decompress and provided a distraction from work. As described by one of the respondents: “There are resources in the office, PlayStation, games, etc. to take our minds off of it.” Varied work hours and flexible schedules provide investigators with a quality of life: “Giving them the flexibility to work a schedule that allows them to have a quality of life.” Finally, specific practices were also put in place in order to mitigate the impact of CSAM investigation. These practices varied by agency, and some were more specific than others. One respondent stated, “Some of the other things we set up within our ICAC that aren't part of our wellness program also helps. Not looking at images within 1 h of leaving. Policies about not listening if watching CSAM. If listening to CSAM no watching”.

Most of the respondents (83.3%) said their agency offered ample vacation/personal time off. Almost half said their agency provided daily opportunities for CSAM investigators to debrief with other CSAM investigators (48.3%) and provided regular administrative updates about positive outcomes from CSAM investigations (41.6%). Each of these were significantly more common among ICAC Task Forces than in affiliated agencies. Other options for personnel that could help promote wellness were common, including having an Employee Assistance Program (76.6%); chaplains (64.1%); an onsite gym (63.6%); outdoor grounds for walking, running or sitting to eat (53.6%), and peer counselors (46.9%).

### Placement of Wellness and Other Agency Needs

When asked to place their agency in terms of wellness needs on a scale of 1–10, respondents said that wellness was an average need (*M* = 5.4, SD = 2.2) with no significant difference between ICAC Task Force and affiliated agencies (Table 3). However, most respondents rated the need for more wellness resources for personnel who view CSAM during investigations as a moderate (40.8%) or high (46.1%) need, with no differences between agency types. The biggest problems identified by respondents included the volume of material/cases (41.6%), not having enough people to conduct investigations (38.3%), and not having enough people to conduct forensic exams (33.0%). ICAC Task Force commanders were more likely than affiliated agency respondents to rate each of these as very big problems. Problems less likely to be rated as “very big” included conflicts or frustrations with prosecutors and judges, obsolete equipment, lack of training for investigating CSAM cases, a general culture of the agency not understanding CSAM investigations, and administrations that did not understand the work they do.

**Table 3:** Needs and challenges to implementing wellness programs.

	**Combined** **(*n =* 209) % (*n*)**	**ICAC Affiliate Agencies** **(*n =* 155) % (*n*)**	**ICAC Task Forces** **(*n =* 54) % (*n*)**	***P*** **-value**
**Placement of your agency on needs associated with wellness (M, SD)**	5.4 (2.2)	5.5 (2.2)	5.2 (2.0)	0.25
**Need for more wellness resources in your agency for personnel who have to view CSAM during investigations**				
Low priority need	13.1 (27)	12.5 (19)	14.8 (8)	0.81
Medium priority need	40.8 (84)	40.1 (61)	42.6 (23)	
High priority need	46.1 (95)	47.4 (72)	42.6 (23)	
**Rated a very big a problem in agency**				
Not enough people to conduct forensic exams	33.0 (69)	26.5 (41)	51.9 (28)	0.001
Not enough people to conduct investigations	38.3 (80)	27.1 (42)	70.4 (38)	<0.001
Volume of material/cases	41.6 (87)	31.6 (49)	70.4 (38)	<0.001
Obsolete equipment	7.2 (15)	8.4 (13)	3.7 (2)	0.25
Lack of training for investigating CSAM cases	5.3 (11)	5.8 (9)	3.7 (2)	0.55
Conflicts or frustrations with the way prosecutors handle CSAM cases	9.1 (19)	7.7 (12)	13.0 (7)	0.25
Conflicts or frustrations with the way judges handle CSAM	20.6 (43)	17.4 (27)	29.6 (16)	0.06
General culture of agency does not understand CSAM investigations	10.1 (21)	9.0 (14)	13.0 (7)	0.41
Administration does not understand the work we do	13.9 (29)	12.9 (20)	16.7 (9)	0.49

### Characteristics of Agencies Engaging in More Proactive Efforts Around Wellness

We examined a variety of ways agencies were engaging in more proactive efforts to help ensure wellness among their personnel who work CSAM cases. First, characteristics associated with agencies who *give personnel preparation before CSAM exposure* included being an ICAC Task Force (vs. affiliated agency), the availability of mental health services within the agency, a lot of respect given to personnel who investigated sex crimes, and having union support for wellness, while holding other possible explanatory factors equal (Table 4). Second, *screening personnel for child abuse and neglect experience* was more common in agencies where a lot of respect was given to personnel who investigated sex crimes, where there was union support for wellness, where the participant was very concerned about the impact of viewing CSAM, and where the participant had received training on CSAM.

**Table 4:** Characteristics of agencies engaging in more proactive effort around wellness—policies and practices.

	**Personnel given preparation before CSAM exposure**		**Personnel screened for child abuse and neglect experience**	
	**Unadjusted OR**	**Adjusted OR**	**Unadjusted OR**	**Adjusted OR**
**Agency characteristic**				
ICAC Task Force (vs affiliate)	6.3 (2.8,14.2)^***^	5.4 (1.9,15.6)^**^	3.4 (1.7,6.8)^***^	1.9 (0.8,4.4)
Forensic examiners in house	3.2 (1.8,5.8)^***^	1.9 (0.9,3.8)	1.5 (0.9,2.7)	—
In house mental health services	4.9 (1.3,18.5)^*^	4.7 (1.0,22.0)^*^	4.5 (1.2,17.0)^*^	3.8 (0.9,16.0)
Very acceptable for personnel to seek mental health help in agency	2.2 (1.3,3.9)^**^	1.0 (0.5,2.0)	2.5 (1.4,4.3)^**^	1.4 (0.7,2.8)
A lot of respect given to personnel who investigate sex crimes in agency	2.5 (1.4,4.3)^**^	2.0 (1.0,3.8)^*^	3.7 (2.1,6.6)^***^	3.1 (1.6,5.9)^***^
Utilizes a CAC	0.8 (0.3,2.3)	—	1.6 (0.6,4.3)	—
Union support for wellness	2.9 (1.6,5.4)^***^	3.7 (1.6,8.7)^**^	2.5 (1.4,4.5)^**^	2.3 (1.1,4.5)^*^
No union	0.4 (0.2,0.8)^**^	1.6 (0.7,3.8)	0.8 (0.4,1.5)	—
**Participant characteristic**				
Female participant	0.9 (0.4, 1.8)	—	1.0 (0.5,1.9)	—
Very concerned about impact of viewing CSAM	2.3 (1.2,4.1)^**^	1.3 (0.6,2.6)	3.4 (1.8,6.3)^***^	2.6 (1.2,5.4)^**^
Attended training on CSAM	2.1 (1.1,3.7)^**^	1.5 (0.8,2.9)	2.6 (1.4,4.6)^**^	2.0 (1.0,3.9)^*^
Number of types of problems seen among personnel	1.1 (1.0,1.3)	—	1.2 (1.0,1.3)^**^	1.1 (0.9,1.2)
Placement of wellness needs	0.9 (0.8,1.0)	—	0.9 (0.8,1.0)	—

*** *p < 0.001*,

** *p < 0.01*,

* *p < 0.05*.

*OR, odds ratio*.

Third, having *CSAM support policies in place*, such as group or individual sessions led by a mental health professionals where reactions to CSAM were discussed, was more common in ICAC Task Forces, in agencies where it was very acceptable for personnel to seek mental health help, and where there was union support for wellness (Table 5). Finally, having an *Officer Wellness Program* was more common in agencies where there was union support for wellness and availability of mental health services within the agency.

**Table 5:** Characteristics of agencies engaging in more proactive effort around wellness—resources and programs.

	**Agency CSAM support policies**		**Officer wellness program**	
	**Unadjusted OR**	**Adjusted OR**	**Unadjusted OR**	**Adjusted OR**
**Agency characteristic**				
ICAC Task Force (vs affiliate)	39.3 (5.3,291.5)^***^	32.9 (4.0,269.1)^***^	1.3 (0.7,2.5)	—
Forensic examiners in house	2.0 (1.1,3.6)^*^	0.8 (0.4,1.6)	1.7 (0.9,3.0)	—
In house mental health services	2.6 (0.9,8.2)	—	6.1 (1.6,22.9)^**^	5.5 (1.4,21.5)^**^
Very acceptable for personnel to seek mental health help in agency	4.7 (2.5,8.8)^***^	2.8 (1.4,5.7)^**^	1.9 (1.1,3.4)^*^	1.5 (0.8,2.9)
A lot of respect given to personnel who investigate sex crimes in agency	2.8 (1.5,5.2)^***^	1.9 (1.0,3.9)	1.2 (0.7,2.1)	—
Utilizes a CAC	0.6 (0.2,1.8)	—	0.3 (0.1,1.1)	—
Union support for wellness	2.5 (1.3,4.7)^**^	2.5 (1.0,6.1)^*^	3.5 (1.8,6.7)^***^	3.2 (1.6,6.3)^***^
No union	0.5 (0.2,0.9)^*^	1.3 (0.5,3.0)	0.6 (0.3,1.1)	—
**Participant characteristic**				
Female participant	1.0 (0.5,2.0)	—	0.6 (0.3,1.2)	—
Very concerned about impact of viewing CSAM	2.7 (1.4,5.3)^**^	1.8 (0.8,4.0)	1.1 (0.6,1.9)	—
Attended training on CSAM	1.3 (0.7,2.4)	—	0.8 (0.4,1.5)	—
Number of types of problems seen among personnel	1.1 (0.9,1.3)	—	1.1 (1.0,1.3)	—

*** *p < 0.001*,

** *p < 0.01*,

* *p < 0.05*.

*OR, odds ratio*.

## Discussion

This study gathered information from leaders of agencies that investigate CSAM about their agency's practices and policies to mitigate trauma and promote resilience among investigators. The results show concern about the problem and a diffusion of proactive initiatives, but still large gaps, barriers, and inconsistent adoption remain. The gaps are particularly noticeable between the specialty oriented ICAC Task Forces and their less-specialized, affiliated agency partners.

### The ICAC Task Force Program Has Taken Steps to Promote Wellness

Exposure to CSAM as part of investigations or forensic work may be a source of stress for some personnel and the findings from this study suggest that ICAC Commanders and leads in affiliated agencies are aware of this potential. Indeed, wellness resources were available to investigators exposed to CSAM in a majority of agencies. Six in 10 respondents overall said their agencies had an Officer Wellness Program. Most agencies held staff training sessions that included discussion of stress at work, at home, and relationship problems. Notably, seven in 10 said trainees were given the option to self-select out of CSAM investigations once they had seen some of the material.

### Challenges and Barriers to Wellness Remain

The study also highlighted considerable gaps in protective practices, however. Almost half (46.1%) of respondents mentioned that the need for more wellness resources in their agency for personnel who had viewed CSAM was a high priority. Most agencies did not provide regular check-ups with mental health professionals, few allowed for part time assignments, and exit tickets were not routine. A sizeable minority of agencies highlighted difficult working conditions; frequently mentioned barriers included having too much material to review and inadequate staffing. Most agencies said that debriefing about cases and updating about positive outcomes were not part of standard practice.

Another practice that was also not standard, present in only one-quarter of all agencies, was mandatory participation in wellness programs. Some advocates believe such mandates are important to overcome the stigma of receiving check-ups or help (29). But other leaders felt that mandates would be resented by investigators, waste staff time, and were not necessary for most staff.

Stigma created by help-seeking was the most widely acknowledged barrier discussed in relation to police wellness. As one respondent noted: “although progress has been made, there is still a long way to go until mental health seeking is understood and widely accepted.” Our findings correspond with previous studies indicating only a minority (9.3%) of law enforcement investigators and digital forensic examiners working on child exploitation cases sought counseling/treatment (5). This could be a true lack of need, but it may also reflect the so-called “neutral attitudes” law enforcement officers have toward seeking professional services (30). Law enforcement officers can be reluctant to seek treatment for fear of being seen as weak or unfit for duty (30). This is especially important given law enforcement personnel can be unaware of burnout and vicarious trauma (31). Some contend that if mental health services are not mandated, law enforcement personnel are unlikely to seek professional help for work-related stress. It was suggested by respondents that making participation in a wellness program mandatory could go a long way in this regard by removing some of the stigma associated with attendance. Mandatory participation would essentially “blind” colleagues from knowing which personnel are struggling at any given time. Likely, such mandates will not be implemented without more incentive, such as research evidence for their positive impact on staff retention and wellness or requirements tied to federal funding.

Another inconsistent practice across agencies was screening requirements for new examiners and investigators. For example, it was generally not required or prioritized for candidates to have had previous work with child abuse cases. Specific mental health testing or screening of potential CSAM investigators was also rare, although several respondents noted that this was part of the police hiring process more generally.

Open-ended responses indicated that certain other capacities were key to hiring personnel for these cases—technical expertise and forensics, personality traits considered important for this work and a willingness to work these types of cases. Clearly there is no consensus yet about whether special screening beyond standard police hiring protocols is useful or beneficial for those tasked to work with CSAM. The argument for special screening for these workers is that they face exposures that are unusual and typically unanticipated by those considering police careers, and that particular life experience and conditions, like a history of abuse or parenting vulnerable children, may predispose them to negative reactions to these specific exposures. There is little research evidence yet to support these concerns. Screening police to detect vulnerability to mental health has not had particular success (32). Moreover, the development of helpful screening tools may be challenging. Police have been shown to be reluctant to be candid on screening tests, which may be one reason for the failures of prediction (33, 34). Nonetheless, this question of how to choose and prepare investigators should remain a top priority of those concerned with wellness.

Not surprisingly, funding was a key concern among agency leaders, ranging from challenges finding funding, limited funding, and inconsistent funding. One respondent even noted that wellness was “always the last thing to be funded.” Although Officer Wellness Programs are common, they are not universal. As such, priority should be given to ensure consistent and inclusive funding for wellness so personnel can stay healthy and feel supported in their work.

### More Wellness Support and Development Are Needed for ICAC Affiliated Agencies

In accounting for gaps in practice, a large and salient problem was the persistent lack of wellness practices in the affiliated agencies in comparison to the Task Forces themselves. Specifically, affiliated agencies were much less likely to have meetings and/or trainings on signs of stress, opportunities to debrief with mental health professionals or peers, or opportunities to transfer should the burden of CSAM investigations become overwhelming or otherwise unhealthy. This is somewhat concerning given that personnel in affiliated agencies may not receive any or only limited preparation before they are first exposed to CSAM.

The gap in affiliated agency practices may stem from a number of factors. They may have fewer staff dedicated to CSAM investigations. They may have leadership that has multiple responsibilities besides CSAM. They may conduct fewer CSAM investigations overall. Central to the difference may be the Task Forces' receipt of more funding for CSAM-specific activities. The findings suggest that funders and policy makers need to prioritize affiliated agency practice development. This could be in the form of enhanced requirements, additional training, and supplemental funding to achieve parity with Task Force practices.

Another major predictor of health-promotion practices was union support. Agencies with union support for wellness had a two- to three-fold greater likelihood of proactive programs or practices. The role of police unions has been a neglected component in research about many aspects of policing, even given their recognition as having a critical role in daily police work (35). Police unions may influence agency culture around wellness and help-seeking, but more research is needed to better understand their role. Findings also highlight the priority need to provide alternative sources of training and motivation to leaders in non-union agencies.

### Recommendations

The results of this study suggest several key recommendations for ICAC Task Forces and their affiliated agencies in helping to improve resilience and promote wellness among personnel who participate in investigations involving CSAM:

1. Promote inter- and intra-agency culture that actively supports the mental health and wellness of ICAC investigators and forensic examiners.
2. Continue to train more police and forensic examiners on the investigation of CSAM so more staff can be placed on these investigations to help reduce burden due to lack of staffing resources.
3. Include police wellness awareness as a priority for incoming Commanders and department leads so they can better support their team and promote strategies for wellbeing.
4. Provide more preparation and awareness for new staff who are working CSAM cases around the possible mental health impact of the job and strategies to promote wellbeing.
5. Make Officer Wellness Programs an integral part of ICAC Task Force funding. Supplementing the standard employee assistance programs and mental health access with more specific information about warning signs and mitigation strategies surrounding CSAM investigations is a good first step.
6. Provide more and easy access to known strategies that help investigators stay healthy while at work including flexible work hours, exercise opportunities, and friend and family events.
7. Provide special focus on the development of practices and resources in affiliated agencies and agencies without supportive unions. These appear to be two markers of less adoption of wellness practices.
8. Direct more training to administrative staff. The research suggests they have exposure to CSAM, but they may be even less prepared for such and more vulnerable than staff with formal police and investigative training (36).
9. Consider how a combination of factors, including not only exposure to the content but also the volume of work, contributes to stress and trauma.

### Limitations

Although we spoke to the majority of ICAC Commanders, the sample of affiliated agencies in this study was much smaller than the overall number of such agencies given we relied on the nomination of the ICAC Commanders for affiliated agency participation (we did not have contact information for all affiliated agencies). As such, the findings should not be interpreted to reflect the experiences of all ICAC affiliated agencies, nor all law enforcement agencies across the United States. This study was designed to address wellness around the exposure to CSAM specifically and less about other associated stressors, like resources and workload. Research inclusive of a broader range of stressful police work would have provided more context to the practices and policies in these agencies. This research was conducted at one point in time and did not take into account how long the specific Task Forces had been in operation, nor how long the Commanders had been in their positions; both of these factors have implications for the development and utilization of wellness programs in these agencies.

## Conclusion

The findings from this paper provide important information regarding our understanding of how agencies who investigate sex crimes against children can support their personnel and help promote wellbeing. Exposure to CSAM can be a source of stress for personnel and the results show concern about the problem and a diffusion of proactive initiatives, but still large gaps, barriers, and inconsistent adoption remain.

## Data Availability Statement

The raw data supporting the conclusions of this article will be made available by the authors, without undue reservation.

## Ethics Statement

The studies involving human participants were reviewed and approved by University of New Hampshire Institutional Review Board. Written informed consent for participation was not required for this study in accordance with the national legislation and the institutional requirements.

## Author Contributions

KM, AG-M, and DF contributed toward instrument development. AG-M and JO'B conducted the qualitative coding while KM analyzed the quantitative data. KM was primarily responsible for the writing of the manuscript. AG-M, JO'B, and DF provided critical review and editing. All authors contributed to the article and approved the submitted version.

## Funding

All phases of this study were supported by a National Institute of Justice grant [2019-R2-CX-0034].

## Author Disclaimer

Opinions or points of view expressed on this manuscript represent a consensus of the authors and do not necessarily represent the official position or policies of the U.S. Department of Justice.

## Conflict of Interest

The authors declare that the research was conducted in the absence of any commercial or financial relationships that could be construed as a potential conflict of interest.

## Publisher's Note

All claims expressed in this article are solely those of the authors and do not necessarily represent those of their affiliated organizations, or those of the publisher, the editors and the reviewers. Any product that may be evaluated in this article, or claim that may be made by its manufacturer, is not guaranteed or endorsed by the publisher.

## References

[1] Bursztein E Bright T Clarke E DeLaune M Eliff DM Hsu N. Rethinking the detection of child sexual abuse imagery on the Internet. In: 2019 World Wide Web Conference (WWW '19). ACM, New York, NY. p. 7. 10.1145/3308558.3313482

[2] Wortley R Smallbone S Powell M Cassematis P. (2014). Understanding and Managing the Occupational Health Impacts on Investigators of Internet Child Exploitation.

[3] National Center for Missing Exploited Children (2021). CyberTipline. Alexandria, VA. Available online at: https://www.missingkids.org/gethelpnow/cybertipline (accessed October 27, 2021).

[4] Sinclair R Duval K Fox E. Strengthening Canadian law enforcement and academic partnerships in the area of online child sexual exploitation: the identification of shared research directions. Child Youth Serv. (2015) 36:345–64. 10.1080/0145935X.2015.1096588

[5] Seigfried-Spellar KC. Assessing the psychological well-being and coping mechanisms of law enforcement investigators vs. digital forensic examiners of child pornography investigations. J. Police Crim.Psychol. (2018) 33:215–26. 10.1007/s11896-017-9248-7

[6] Brown J Fielding J Grover J. Distinguishing traumatic, vicarious and routine operational stressor exposure and attendant adverse consequences in a sample of police officers. Work Stress. (1999) 13:312–25. 10.1080/02678379950019770

[7] Gershon RR Barocas B Canton AN Li X Vlahov D. Mental, physical, and behavioral outcomes associated with perceived work stress in police officers. Crim Justice Behav. (2009) 36:275–89. 10.1177/009385480833001526364008

[8] Burruss GW Holt TJ Wall-Parker A. The hazards of investigating internet crimes against children: Digital evidence handlers' experiences with vicarious trauma and coping behaviors. Am. J. Crim. Just. (2018) 43:433–47. 10.1007/s12103-017-9417-3

[9] Anderson W Bauer B. Law enforcement officers: The consequences of exposure to violence. J. Counsel. Develop. (1987) 65:381–4. 10.1002/j.1556-6676.1987.tb00736.x19943932

[10] Follette VM Polusny MM Milbeck K. Mental health and law enforcement professionals: trauma history, psychological symptoms, and impact of providing services to child sexual abuse survivors. Prof. Psychol. Res. Pract. (1994) 25:275. 10.1037/0735-7028.25.3.275

[11] Van Patten IT Burke TW. Critical incident stress and the child homicide investigator. Homicide Stud. (2001) 5:131–52. 10.1177/1088767901005002003

[12] Violanti JM Gehrke A. Police trauma encounters: precursors of compassion fatigue. Int J Emerg Ment Health. (2004). 15298078

[13] Moulden HM Firestone P. Vicarious traumatization: the impact on therapists who work with sexual offenders. Trauma Violence Abuse. (2007) 8:67–83. 10.1177/1524838006297729 17204600

[14] Krause M. Identifying and managing stress in child pornography and child exploitation investigators. J. Police Crim. Psychol. (2009) 24:22–9. 10.1007/s11896-008-9033-8

[15] Perez LM Jones J Englert DR Sachau D. Secondary traumatic stress and burnout among law enforcement investigators exposed to disturbing media images. J. Police Crim. Psychol. (2010) 25:113–24. 10.1007/s11896-010-9066-7

[16] Powell M Cassematis P Benson M Smallbone S Wortley R. Police officers' perceptions of their reactions to viewing internet child exploitation material. J. Police Crim. Psychol. (2015) 30:103–11. 10.1007/s11896-014-9148-z

[17] Burns R Loh V Byles J Kendig H. The impact of childhood parental quality on mental health outcomes in older adults. Aging Mental Health. (2018) 22:819–25. 10.1080/13607863.2017.1317331 28436695

[18] Burns CM Morley J Bradshaw R Domene J. The emotional impact on and coping strategies employed by police teams investigating internet child exploitation. Traumatology. (2008) 14:20–31. 10.1177/1534765608319082

[19] Wolak J Mitchell KJ. Work Exposure to Child Pornography in ICAC Task Forces and Affiliates (2009). Durham, NH: Crimes against Children Research Center, University of New Hampshire.

[20] Evans BJ Coman GJ Stanley RO Burrows GD. Police officers' coping strategies: an Australian police survey. Stress Med. (1993) 9:237–46. 10.1002/smi.2460090406

[21] Hart PM Wearing AJ Headey B. Police stress and well-being: Integrating personality, coping and daily work experiences. J Occup Organ Psychol. (1995) 68:133–56. 10.1111/j.2044-8325.1995.tb00578.x

[22] Brough P. Comparing the influence of traumatic and organizational stressors on the psychological health of police, fire, and ambulance officers. Int J Stress Manag. (2004) 11:227. 10.1037/1072-5245.11.3.227

[23] Bokelberg GA. Stress associated with investigating and working in support of investigations of internet sexual crimes against children. In: Unpublished study. (2005). Washington, DC: FBI.

[24] Cohen IM McCormick AV Rich B. Creating a culture of police officer wellness. Policing J Policy Pract. (2019) 13, 213–229. 10.1093/police/paz001

[25] Shochet IM Shakespeare-Finch J Craig C Roos C Wurfl A Hoge R. The development and implementation of the Promoting Resilient Officers (PRO) program. Traumatol Int J. (2011) 17:43–51. 10.1177/1534765611429080

[26] International Association of Chiefs of Police. Officer Health and Wellness. Agency Assessment Tool and Action Planning Roadmap. (2021). Washington, DC: Bureau of Justice Assistance, U.S. Department of Justice.

[27] Graneheim UH Lundman B. Qualitative content analysis in nursing research: concepts, procedures and measures to achieve trustworthiness. Nurse Educ Today. (2004) 24:105–12. 10.1016/j.nedt.2003.10.001 14769454

[28] Hsieh H-F Shannon SE. Three approaches to qualitative content analysis. Qual Health Res. (2005) 15:1277–88. 10.1177/1049732305276687 16204405

[29] Jetelina KK Molsberry RJ Gonzalez JR Beauchamp AM Hall T. Prevalence of mental illness and mental health care use among police officers. JAMA Netw. Open. (2020) 3:e2019658–e2019658. 10.1001/jamanetworkopen.2020.19658 33026452 PMC7542299

[30] Karaffa KM Tochkov K. Attitudes toward seeking mental health treatment among law enforcement officers. Appl Psychol Crim Justice. (2013) 9:75–99.

[31] Slack DP. Trauma and coping mechanisms exhibited by forensic science practitioners: a literature review. Forensic Sci Int. Synergy. (2020) 2:310–6. 10.1016/j.fsisyn.2020.10.001 33163953 PMC7606841

[32] Marshall R Milligan-Saville J Steel Z Bryant R Mitchell P Harvey S. A prospective study of pre-employment psychological testing amongst police recruits. Occup Med. (2020) 70:162–8. 10.1093/occmed/kqaa008 32040153

[33] Bell S Palmer-Conn S. Suspicious minds: Police attitudes to mental ill health. Int. J. Law Public Admin. (2018) 1:25–40. 10.11114/ijlpa.v1i2.3878

[34] Marshall RE Milligan-Saville J Petrie K Bryant RA Mitchell PB Harvey SB. Mental health screening amongst police officers: factors associated with under-reporting of symptoms. BMC Psychiatry. (2021) 21:1–8. 10.1186/s12888-021-03125-1 33685431 PMC7938555

[35] Walker S. The neglect of police unions: exploring one of the most important areas of American policing. Police Pract Res Int J. (2008) 9:95–112. 10.1080/15614260802081253

[36] Lentz L Silverstone PH Krameddine YI. High rates of mental health disorders in civilian employees working in police organizations. Front Psychol. (2020) 11:1031. 10.3389/fpsyg.2020.01031 32547453 PMC7270335

